# Compliance with Iron-Folate Supplement and Associated Factors among Antenatal Care Attendant Mothers in Misha District, South Ethiopia: Community Based Cross-Sectional Study

**DOI:** 10.1155/2015/781973

**Published:** 2015-12-29

**Authors:** Abinet Arega Sadore, Lakew Abebe Gebretsadik, Mamusha Aman Hussen

**Affiliations:** ^1^Hadiya Zone Health Department, Southern Nations, Nationalities and Peoples, Ethiopia; ^2^Department of Health Education and Behavioral Sciences, Jimma University, P.O. Box 378, Jimma, Ethiopia

## Abstract

*Background.* In Ethiopia, higher proportions of pregnant women are anemic. Despite the efforts to reduce iron deficiency anemia during pregnancy, only few women took an iron supplement as recommended. Thus, this study aimed to assess compliance with iron-folate supplement and associated factors among antenatal care attendant mothers in Misha district, South Ethiopia.* Method*. Community based cross-sectional study supported with in-depth interview was conducted from March 1 to March 30, 2015. The sample size was determined using single population proportion to 303. Simple random sampling technique was used to select the study participants. Bivariate and multivariable logistic regression analyses were employed to identify factors associated with compliance to iron-folate supplement.* Results*. The compliance rate was found to be 39.2%. Mothers knowledge of anemia (AOR = 4.451, 95% CI = (2.027,9.777)), knowledge of iron-folate supplement (AOR = 3.509, 95% CI = (1.442,8.537)), and counseling on iron-folate supplement (AOR = 4.093, 95% CI = (2.002,8.368)) were significantly associated with compliance to iron-folate supplement.* Conclusions.* Compliance rate of iron-folate supplementation during pregnancy remains very low. This study showed that providing women with clear instructions about iron-folate tablet intake and educating them on the health benefits of the iron-folate tablets can increase compliance with iron-folate supplementation.

## 1. Background

The World Health Organization estimates the number of anemic people worldwide to be very high (2 billion) and that approximately 50% of all anemia can be attributed to iron deficiency [1]. Currently, the global prevalence of anemia is estimated to be 30.2% in nonpregnant women rising to 41.8% during pregnancy. Anemia prevalence among pregnant women is around 24.1% in the Americas, 48.2% in South East Asia, 25.1% in Europe, 44.2% in Eastern Mediterranean, 30.7% in the Western Pacific, and the highest in Africa at 57.1% [2–4]. In Ethiopia, higher proportion of pregnant women are anemic (22%) than women who are breastfeeding (19%) [5]. Though anemia has multifaceted causes, half of its burden is attributed to iron deficiency (ID) [6]. According to the World Health Organization (WHO), 12.8% and 3.7% of maternal mortality in Asia and Africa, respectively, are directly attributable to anemia [7]. The prevalence of anemia in pregnancy has remained unacceptably high worldwide, especially in developing countries over the past three decades [1]. The World Health Organization has recommended a 6-month regimen of a daily supplement containing 60 mg of elemental iron along with 400 *μ*g of folic acid for all pregnant mothers. In areas with a higher prevalence of anemia, it is recommended that supplementation continues for three months postpartum [1]. Accordingly, in Ethiopia, the national guideline for control and prevention of micronutrient deficiencies highlights the need of daily iron supplementation for at least 6 months during pregnancy and 3 months postpartum [8]. Despite this program, in Ethiopia, <1% took an iron supplement for the recommended period (90 days or more) during their last pregnancy [5].

Therefore, the major problem with iron-folate supplementation in pregnancy is compliance, as women often fail to take the supplements regularly as supplemented by their health workers due to varying factors [9, 10] and this is thought to be a potential driver to the persistent high prevalence of anemia in pregnant mothers. This emphasizes the need to study the factors influencing compliance with iron-folate supplements.

*Objective of the Study*

 General objectives:
  - To assess compliance with iron-folate supplement and associated factors among antenatal care attendant mothers in Misha district, South Ethiopia, 2015.
 Specific objectives:
  - To determine status of compliance with iron-folate supplement among ANC attendant mothers.
  - To identify factors affecting compliance with iron-folate supplement among ANC attendant mothers.

## 2. Methods and Materials

### 2.1. Study Setting and Design

A community based cross-sectional study using quantitative and qualitative data collection methods was conducted from March 1 to March 10, 2015, among randomly selected pregnant mothers in Misha district. Misha is one of the districts in the Hadiya zone of Southern Ethiopia. It has a total of 5,537 pregnant women. There are seven health centers and thirty-five health posts, which provide routine antenatal care services to the community.

### 2.2. Study Populations

The study population consisted of sampled pregnant mothers in the study area who were attending ANC and being supplied with iron-folate supplement. Pregnant mothers who did not participate in quantitative study and health extension workers were participants of qualitative study. Pregnant mothers who had stayed in antenatal care for at least one month preceding the study period and had received iron-folate supplementation were included in the current study. However, those who were unable to respond or very sick were excluded.

### 2.3. Sample Size and Sampling Procedures

The sample size was determined based on the single population proportion formula using *Z*_2_ × *p* × *q*/*d* ^2^ with a 95% CI, 5% margin of error, and an assumption that 74.9% of pregnant women are compliant with iron-folate supplement in the study area [11]. Assuming a 10% nonresponse rate, a total sample size of 303 pregnant women was required. A total of 13 participants, including 8 pregnant mothers who were not involved in quantitative study and 5 health extension workers, participated in in-depth interview.

Eleven kebeles were selected using a lottery method from thirty-five kebeles. A proportional allocation was employed to obtain the sample size of the selected kebeles. Prior to the actual data collection, the list of study subjects was identified by using community health management information system (CHMIS) folder. Finally, the study participants were selected by using random numbers generated by a computer program. The criterion purposive sampling technique was used to select pregnant mothers and health extension workers for qualitative study.

### 2.4. Data Collection

Pretested and structured questionnaire was used to collect quantitative data. The questionnaire was designed in English and translated into Hadiyisa (a local language). In order to ensure that the original meanings of the items on the questionnaire were maintained, the Hadiyisa version was translated back into English by a researcher conversant in both languages. The two versions were examined to identify any inconsistency in the wording. The final Hadiyisa version was pretested on a sample of 15 pregnant mothers attending ANC and using IFA supplement in neighbor district or outside the study area. The questionnaire consisted of items that assessed sociodemographic characteristics, compliance to supplements, knowledge of anemia and iron-folate supplement, pregnancy and related experiences, and facility and supplement related factors. An interview guide was used for in-depth interviews.

### 2.5. Study Variables

The outcome for this study, compliance to IFA supplement, was assessed based on the reported number of IFA tablets taken in the previous 7 days before the survey. Pregnant mothers who took at least 70% of the expected dose of the iron-folate tablets in the previous week before the study, which is equivalent to consuming at least five tablets per week, were considered as compliant with iron-folate supplement. The respondents who consumed less than five IFA tablets were considered as noncompliant [11]. The independent variables include respondent's sociodemographic characteristics (age, educational status, occupation, and income), pregnancy and related experience (gravidity, parity, and frequency of ANC visits), health care related factors (distance to the nearest health facility, counseling about IFA), and knowledge of anemia and IFA supplement. Mother age was categorized into three groups by five-year interval, which was later recoded into two categories as <25 and ≥25 because they were very few cases per cell to run logistic regressions. Similarly, maternal occupation was categorized into three later recoded into two categories. The respondents' comprehensive knowledge of anemia was computed by summing up 18 multiple-choice items (4 items on the sign and symptoms of anemia; 6 items for anemia causes; 6 items for consequences of anemia during pregnancy; and 2 items on prevention and treatment of anemia during pregnancy). Comprehensive knowledge of iron-folate supplement was measured by summing up 8 multiple-choice items. A correct answer was given one mark, while a wrong answer was not given any mark.

### 2.6. Statistical Analysis

Data were entered into and cleaned using Epi data version 3.1 and then exported to SPSS version 16 for further analysis. Descriptive statistics were used to describe the study variables. Chi-squared test was used to determine adequacy of the cells. Then, binary logistic regression was used to examine the relationship between the proposed predictors and compliance to iron-folate supplement. Variables with *p* value ≤0.25 in the bivariate analysis were entered into multivariable logistic regression to identify variables independently associated with iron-folate supplement compliance. 95% CI with a respective odd ratio was used to assess the statistical significance of association among the variables. *p* value less than 0.05 was used as cut-off point to see the presence of statistically significant association. In-depth interviews were audio-taped and verbatim-transcribed into Hadiyisa and then directly translated into English. Subsequently, thematic analyses of the data were undertaken.

### 2.7. Ethical Considerations

Ethical clearance and approval for the study was obtained from the ethical committee of Jimma University, College of Health Science. The research presents no more than minimal risk of harm to subjects. Thus, oral consent was obtained from all the respondents after explaining of the purpose of the study, risk/discomfort, benefits to the subject, confidentiality of records, right to refuse participation and terminate participation in the study at any time. The consent process was approved by the ethical committee of Jimma University.

## 3. Results

### 3.1. Socioeconomic and Demographic Characteristics of the Pregnant Women

From a total of 303 pregnant mothers, 296 were involved in the study, yielding a response rate of 97.6%. The mean age of the study participants was 29.07 (SD ± 6.0) years. The rural dweller constituted the major proportion (91.2%). Concerning maternal education, 46.3% had a primary level of education followed by secondary and above (39.2%). The majority of the mothers' occupation was housewife (88.20%) (Table 1).

### 3.2. Compliance with Iron-Folate Supplementation

One hundred eighty (60.8%) respondents took <70% of expected doses of the IFA supplements, that is, for less than five days in a week, and 116 (39.2%) respondents took greater than or equal to 70% of expected doses of the IFA supplement in a week, that is, for greater than or equal to five days in a week. Amongst women who missed the doses of IFA supplement, the leading underlying reason was side effects (125, 50.6%). Of those respondents who reported missing of IFA doses due to side effects, 96 (76.8%) respondents reported heart burn, 19 (15.2%) respondents reported nausea and vomiting, and 10 (8%) respondents reported stomach cramping. The other reasons of skipping doses of IFA supplement were forgetfulness (104, 42.1%), perceived shortage of iron-folate supplements in health facility (13, 5.3%), and not liking the taste of IFA supplement (5, 2%).

Finding from qualitative part of the study revealed that most pregnant mothers' main reason of missing dose of iron-folate supplement was fear of side effects of iron-folate tablet.

*…I feel nausea and heartburn as soon as I took the tablets. So, I stopped to take iron-folate tablets. [Pregnant mothers, age 32]*

Another qualitative finding revealed that the second main reason forwarded by the in-depth interview participants was forgetfulness.

*…I want to take the iron-folate tablet as prescribed, but I forget to take the tablet regularly. [Pregnant mothers, age 26]*

From the key informant interview, most of the participants argued that the main reason for missing the dose of iron-folate supplement was gastrointestinal side effect of the iron-folate tablets “*heartburn.*”

*…Some pregnant mothers reported the missing of doses of iron-folate tablets during their ANC visits the reason they told me that they felt the heartburn (gastric problem) when they took the tablets. [Health extension worker, age 25].*

### 3.3. Comprehensive Knowledge of Anemia

Comprehensive knowledge of anemia was computed from summing up all relevant 18 knowledge items (4 items on sign and symptoms of anemia; 6 items for anemia causes; 6 items for consequences of anemia during pregnancy; and 2 items on prevention and treatment of anemia during pregnancy). A correct answer for each item was scored as “1” and incorrect answer was scored as “0.” Items were then summed up and converted to 100%. Accordingly, the median score was 61.1, mode was 72.2, and the mean was 61.9 (SD = 17.4).

About 56.1% of the respondents scored above the median value; therefore, they had good knowledge of anemia and the remaining scored below the median value.

### 3.4. Comprehensive Knowledge of Iron-Folate Supplement

Comprehensive knowledge of iron-folate supplement was computed by summing up all relevant 8 items (3 items on benefits of iron-folate supplementation and 5 items on possible effects of iron deficiency anemia during pregnancy). A correct answer for each item was scored as “1” and incorrect answer was scored as “0.” Items then were summed up and converted to 100%. Accordingly, the median score was 62.5, mode was 75, and the mean was 63.8 (SD = 21.9).

About 61.5% of the respondents scored above the median value and the remaining scored below the median value.

### 3.5. Pregnancy and Related Experiences

From total respondents, 261 (88.2%) of mothers were multigravida and 35 (11.8%) were primigravida. A high percentage of the pregnant mothers interviewed were in their third trimester (239, 80.7%) whereas 57 (19.3%) were in their second trimester. Concerning the parity of the respondents, 191 (64.5%) of mothers were multipara and 66 (22.3%) and 39 (13.2%) of respondents were primipara and nullpara, respectively. Of all respondents, 55 (18.6%) had a history of anemia during pregnancy confirmed by clinical health workers. Concerning the utilization of antenatal care, 261 (88.2%) of respondents visited ANC for less than four times and 35 (11.8%) of respondents visited ANC for greater than or equal to four times.

### 3.6. Supplement Related Variable

As part of supplement related variable, only 5 (1.6%) respondents reported the disliking of taste of iron-folate supplement.

### 3.7. Facility Related Variables

Of the total respondents, 232 (78.4%) said that it took them 30 minutes or less to reach the nearest health institution from their residence, 62 (20.9%) of pregnant mothers said that it took 30–60 minutes to reach the institution, and 2 (0.7%) mothers reported it took greater than 60 minutes to reach the nearest health institution. One hundred fifty-four (52%) of respondents got counseling on IFA tablets and 48% of respondents did not get counseling on IFA tablets.

### 3.8. Factors Affecting Compliance with Iron-Folate Supplement

To investigate the association of predictors with IFA supplement, both univariate and multivariable analysis were used. Predictors which showed an association with IFA supplement at *p* value of less than or equal to 0.25 in the univariate were selected as candidate variables for multivariable logistic regression analysis. Among the variables entered to multivariable logistic regression, age of mothers, counseling on iron-folate supplement, knowledge of IFA supplement, knowledge of anemia, and frequency of ANC visits were significantly associated with compliance with iron-folate supplement during pregnancy. Pregnant mothers whose ages were ≥25 years 2.9 times more likely complied with iron-folate supplement than those pregnant mothers who were <25 years old (AOR = 2.985, 95% CI = (1.069,8.340)). Pregnant mothers who had good knowledge of iron-folate supplement were 3.5 times more likely to be compliant with iron-folate supplement as compared to those who had poor knowledge about iron-folate supplement (AOR = 3.509, 95% CI = (1.442,8.537)). Pregnant mothers who had good knowledge of anemia were 4.4 times more likely compliant with iron-folate supplementation as compared to those who had poor knowledge (AOR = 4.451, 95% CI = (2.027,9.777)). Similarly, mothers who had visited ANC four times and above were 3.5 times more likely compliant with IFA supplement as compared to mothers who visited ANC less than four times (AOR = 3.558, 95% CI = (1.189,10.653)). Pregnant mothers who were counseled on iron-folate supplement during pregnancy were 4 times more likely compliant than those who were not counseled on intake of IFA supplement (AOR = 4.093, 95% CI = (2.002, 8.368)) (Table 2).

## 4. Discussion

The result revealed that 39.2% of pregnant mothers were compliant (took at least 70% of the expected dose of the iron-folate tablets in seven days of the previous week of the study) to the supplement, which is much lower compared with the study done in four regions of Ethiopia which was 74.9% [11] and higher than study done in Amhara regions of Ethiopia that was 20.4% [12]. The probable reason may be the difference in geographic locations and the time gap between studies and study subjects.

Even though the compliance rate is low compared with other countries, it is much higher than 0.4% finding of EDHS 2011 [5]. This could be due to differences in level of study (national and district level) and the time gap between the present study and EDHS 2011. The compliance rate among pregnant mothers in this study was still lower compared to studies done in other countries like United State, Philippines, Nigeria, and Senegal [10, 13–15]. This difference may be due to differences in awareness of pregnant mothers about iron-folate supplementation and study design.

The side effect is repeatedly considered as a major problem with compliance. According to studies conducted in the Philippines [14], Senegal [15], and India [16], it was reported as a reason for missing doses of iron-folate supplementation among pregnant mothers by 20.2%, 27.0%, and 27.6%, respectively. Studies conducted in Vientiane [17] also concluded likewise. In this study, it was observed that, from those mothers who missed the doses of IFA supplement, 50.6% were because of fear of side effects. Other studies done in the Amhara region of Ethiopia and eight districts of four regions of Ethiopia revealed that 54.4% and 63.3% of respondents, respectively, missed the dose of iron-folate supplement due to fear of side effects [11, 12]. One probable rationalization for the high figure of side effects in this study can be lack of information about potential side effects of iron-folate supplement in advance. Appropriate counseling is known to increase the psychological tolerance of pregnant mothers to side effects of iron-folate supplement [18].

In this study, 42.1% of pregnant mothers skipped doses of iron-folate supplement because of forgetfulness and this finding is parallel with the findings in India and Vientiane by 48.8% and 47.9%, respectively [13, 17], but it is much higher than the finding in Ethiopia that was 16.7% [11]. The discrepancy may be due to inadequate counseling of mothers to remember to take their tablet in current study. This problem could be addressed through better counseling during the ANC visit by suggesting to women strategies to remember to take their tablets, for example, placing the tablets in a spot that they see every day.

Fear of having big fetus because of consuming iron-folate tablet was obstacle to compliance with iron-folate supplement in previous study which was done in Thailand [19]. One study in Ethiopia also showed that 28.45% of women believed that continuous taking of iron-folate supplementation leads to overweight babies [12]. But, in this study, none of the respondents reported stopping of taking iron-folate supplement because of fear of having big fetus/baby. This could be due to differences in setting of study subjects.

After adjusting for other factors in the regression analysis, one factor shown to have a significant association with the compliance was the participants' age. Women who were ≥25 years old were 2.9 times more likely to be compliant to iron-folate supplementation than women with younger ages (<25 years). The reason for this is that older women may be more concerned about their health and pregnancy outcomes and had better experiences in the prevention and treatment of iron deficiency anemia. This finding was in line with a study in India that elderly and middle women were slightly more compliant than younger women [16] and also this finding is consistent with the study done in Ethiopia [12]. Similarly, visiting ANC four and more times were considered to have significant effects on compliance with iron-folate supplement. Mothers who had visited ANC for four and more times are 3.5 times more likely to be compliant with iron-folate supplement compared to mothers who visited ANC for less than four times. This finding is consistent with finding in Philippines [14]. The possible reason of this is that health providers may help mothers during their ANC visits by discussing compliance with iron-folate supplement, encouraging them to take the tablet as prescribed, and educating them on health benefit of taking IFA supplement, and these help mothers to be compliant with iron-folate supplement.

In this study, pregnant mothers who had good knowledge of anemia were 4.4 times more likely to be compliant with iron-folate supplement during pregnancy compared to those who had poor knowledge. This finding was consistent in Nigeria, Vientiane, and Amhara region of Ethiopia [10, 12, 17]. Pregnant mothers who had good knowledge of iron-folate supplement during pregnancy were 3.5 times more likely to be compliant with iron-folate supplement during pregnancy compared to those who had poor knowledge about iron-folate supplement. Similar finding was found in Nigeria and Amhara region of Ethiopia [10, 12]. The reason could be that knowledge helps women to have a good perception of prevention and treatment of anemia during pregnancy by taking iron-folate supplement during pregnancy.

Pregnant mothers who were counseled on iron-folate supplement during pregnancy 4 times more likely complied than those who were not counseled on IFA supplement. This finding is consistent with the study done in India (Haryana state), Sweden, Cambodia, and Senegal [9, 15, 20, 21].

*Limitation of the Study.* The limitations need to be considered when interpreting the results of this research. Reports of the pregnant mothers may under/overestimate compliance rate since the data was collected by self-report.

## 5. Conclusions and Recommendations

The compliance rate of iron-folate supplementation during pregnancy remains very low in the Misha district. This indicates that the WHO and FMOH recommendations were no met even though there was usefulness of iron-folate supplementation program during pregnancy to prevent iron deficiency and iron deficiency anemia during pregnancy.

In this study, ages of the mothers, counseling on iron-folate supplement, knowledge of anemia, knowledge of iron-folate supplement, and frequency of ANC visits were found to be significantly associated factors of compliance with iron-folate supplementation during pregnancy. Furthermore, fear of side effects of iron-folate supplement, forgetfulness, and perceived shortage of iron-folate supplement in the health facility were commonly mentioned reasons for missing the doses of iron-folate supplement. Therefore, compliance with iron-folate supplementation can be increased by providing women with clear instructions about iron-folate tablet intake and educating them on the anemia and health benefits of the iron-folate tablets. Also, promoting mothers to visit ANC at least four times can improve their status of compliance with iron-folate supplementation.

Recommendation is made based on finding as follows: providing pregnant mothers with clear instructions about iron-folate tablet intake, educating them on anemia during pregnancy and its consequences and health benefits of the iron-folate tablets, and promoting mothers to visit ANC at least four times. Also, further research is recommended on compliance with iron-folate supplement using the pill count method to overcome the limitation of this study.

## Figures and Tables

**Table 1:** Sociodemographic and economic characteristics of respondents in Misha district, Southern Ethiopia, May 2015.

Background variables	Categories	Frequency	Percent
Residence of the mother	Urban	26	8.8
	Rural	270	91.2

Women's educational status	Illiterate	43	14.5
	Primary (1–8)	137	46.3
	Secondary (9–12) and above	116	39.2

Marital status	Single	2	0.7
	Married	291	98.3
	Widowed	3	1.0

Women's occupation	House wife	261	88.2
	Government	25	8.4
	Others^*∗*^	10	3.4

Age of women	15–19	3	1.0
	20–34	222	75.0
	35–49	71	24.0

Monthly income	Low (≤500 Birr)	95	32.1
	Medium (500–900 Birr)	100	33.8
	High (≥900 Birr)	101	34.1

^*∗*^Others include students and self-employed.

**Table 2:** Multivariable logistic regression result of compliance with IFA supplement with independent variables in Misha district, South Ethiopia, May 2015.

Variables	Compliance with IFA supplement		COR (95% CI)	AOR (95% CI)
	Noncompliant	Compliant		
Age of mothers				
<25	49 (76.6)	15 (23.4)		1
≥25	131 (56.5)	101 (43.5)	2.519 (1.336, 4.747)	**2.985 (1.069, **8.340)^*∗*^
Educational status				
No education	33 (76.7)	10 (23.3)		1
Primary (grades 1–8)	98 (71.5)	39 (28.5)	1.313 (0.591, 2.920)	1.227 (0.425, 3.547)
Secondary (grades 9–12) and above	49 (42.2)	67 (57.8)	4.512 (2.032, 10.019)	2.382 (0.838, 6.768)
Occupation				
Housewife	173 (65.3)	92 (34.7)		1
Others	7 (22.6)	24 (77.4)	6.447 (2.677, 15.529)	2.989 (0.773, 11.558)
Counseling on IFA				
Yes	66 (42.9)	88 (57.1)	5.429 (3.220, 9.152)	**4.093 (2.002, **8.368)^*∗*^
No	114 (80.3)	28 (19.7)		1
Knowledge of anemia				
Poor knowledgeable	112 (86.2)	18 (13.8)		1
Good knowledgeable	68 (41.0)	98 (59.0)	8.967 (4.991, 16.112)	**4.451 (2.027, **9.777)^*∗*^
Knowledge of IFA supplement				
Poor knowledgeable	99 (86.8)	15 (13.2)		1
Good knowledgeable	81 (44.5)	101 (55.5)	8.230 (4.442, 15.248)	**3.509 (1.442, **8.537)^*∗*^
Frequency of ANC visits				
<4 times	171 (65.5)	90 (34.5)		1
≥4 times	9 (25.7)	26 (74.3)	5.489 (2.467, 12.214)	**3.558 (1.189, **10.653)^*∗*^
Gravidity				
Primigravida	23 (65.7)	12 (34.3)		1
Multigravida	157 (60.1)	104 (39.9)	1.545 (0.862, 2.766)	1.243 (0.566, 2.729)
Gestational age				
2nd trimester	45 (78.9)	12 (21.1)		1
3rd trimester	135 (56.5)	104 (43.5)	2.889 (1.455, 5.738)	1.432 (0.560, 3.662)
Previous anemia				
Yes	22 (40.0)	33 (60.0)	2.855 (1.565, 5.210)	2.373 (0.982, 5.738)
No	158 (65.6)	83 (34.4)		1
Monthly income				
Low	63 (66.3)	32 (33.7)		1
Medium	61 (61)	39 (39)	0.632 (0.354, 1.128)	0.757 (0.295, 1.940)
High	56 (55.4)	45 (44.6)	0.796 (0.454, 1.395)	0.900 (0.364, 2.225)

^*∗*^Statistically significant at *p* < 0.05 after being adjusted for other variables, 1 = reference.

*Note*. Hosmer and Lemeshow Test = 0.948; therefore, the model adequately fits the data.
